# Supplementary material for: Longitudinal Fecal Microbiota Profiles in A Cohort of Non-Hospitalized Adolescents and Young Adults with COVID-19: Associations with SARS-CoV-2 Status and Long-Term Fatigue
Source: Pathogens. 2024 Oct 31;13(11):953. doi: 10.3390/pathogens13110953 (PMC11597601; doi:10.3390/pathogens13110953)
Supplement: Supplementary file 1 [file pathogens-13-00953-s001.zip › Table S1. Tabell species.pdf]

Acidaminococcus intestini	Bifidobacterium angulatum	Eggerthella lenta	Parabacteroides goldsteinii
Akkermansia muciniphila	Bifidobacterium animalis subsp. lactis	Enterococcus faecalis	Parabacteroides merdae
Akkermansia muciniphila AmI	Bifidobacterium bifidum	Erysipelatoclostridium ramosum	Paraprevotella clara
Alistipes communis	Bifidobacterium catenulatum	Escherichia coli	Parasutterella excrementihominis
Alistipes finegoldii	Bifidobacterium longum	Eubacterium eligens	Parvimonas micra
Alistipes onderdonkii	Bifidobacterium longum subsp. longum	Eubacterium rectale	Phascolarctobacterium faecium
Alistipes putredinis	Bifidobacterium pseudocatenulatum	Eubacterium siraeum	Phocaeicola dorei
Alistipes senegalensis	Bilophila wadsworthia	Eubacterium ventriosum	Phocaeicola massiliensis
Alistipes shahii	Blautia hydrogenotrophica	Faecalibacterium cf. prausnitzii KLE1255	Phocaeicola vulgatus
Anaerostipes hadrus	Blautia obeum	Faecalibacterium prausnitzii	Prevotella copri
Bacteroides caccae	Blautia wexlerae	Faecalibacterium prausnitzii A2-165	Roseburia hominis
Bacteroides cellulosilyticus	Butyrivibrio crossotus	Faecalibacterium prausnitzii AHMP21	Roseburia intestinalis
Bacteroides coprocola	Christensenella minuta	Faecalibacterium prausnitzii CNCM4575	Roseburia inulinivorans
Bacteroides eggerthii	Clostridium bolteae	Faecalibacterium prausnitzii L2-6	Ruminococcus albus
Bacteroides faecis	Clostridium citroniae	Faecalibacterium prausnitzii M21/2	Ruminococcus bicirculans
Bacteroides finegoldii	Clostridium leptum	Flavonifractor plautii	Ruminococcus bromii
Bacteroides fragilis	Clostridium nexile	Gemmiger formicilis	Ruminococcus gnavus
Bacteroides intestinalis	Clostridium scindens	Gordonibacter pamelaeae	Ruminococcus lactaris
Bacteroides nordii	Clostridium spiroforme	Haemophilus parainfluenzae	Ruminococcus torques
Bacteroides ovatus	Clostridium symbiosum	Holdemanella biformis	Streptococcus thermophilus
Bacteroides plebeius	Collinsella aerofaciens	Holdemania filiformis	Subdoligranulum sp. (group 2)
Bacteroides stercoris	Coprococcus catus	Hungatella hathewayi	Subdoligranulum sp. (group 3)
Bacteroides thetaiotaomicron	Coprococcus comes	Intestinibacter bartlettii	Subdoligranulum sp. (group 4)
Bacteroides uniformis	Desulfovibrio piger	Malassezia (genus)	Subdoligranulum variabile
Bacteroides xylanisolvens	Dialister invisus	Methanobrevibacter smithii	Sutterella wadsworthensis
Barnesiella intestinihominis	Dorea formicigenerans	Odoribacter splanchnicus	Veillonella atypica
Bifidobacterium adolescentis	Dorea longicatena	Parabacteroides distasonis	
