# Supplementary figures and images for: Longitudinal Fecal Microbiota Profiles in A Cohort of Non-Hospitalized Adolescents and Young Adults with COVID-19: Associations with SARS-CoV-2 Status and Long-Term Fatigue
Source: Pathogens. 2024 Oct 31;13(11):953. doi: 10.3390/pathogens13110953 (PMC11597601; doi:10.3390/pathogens13110953)

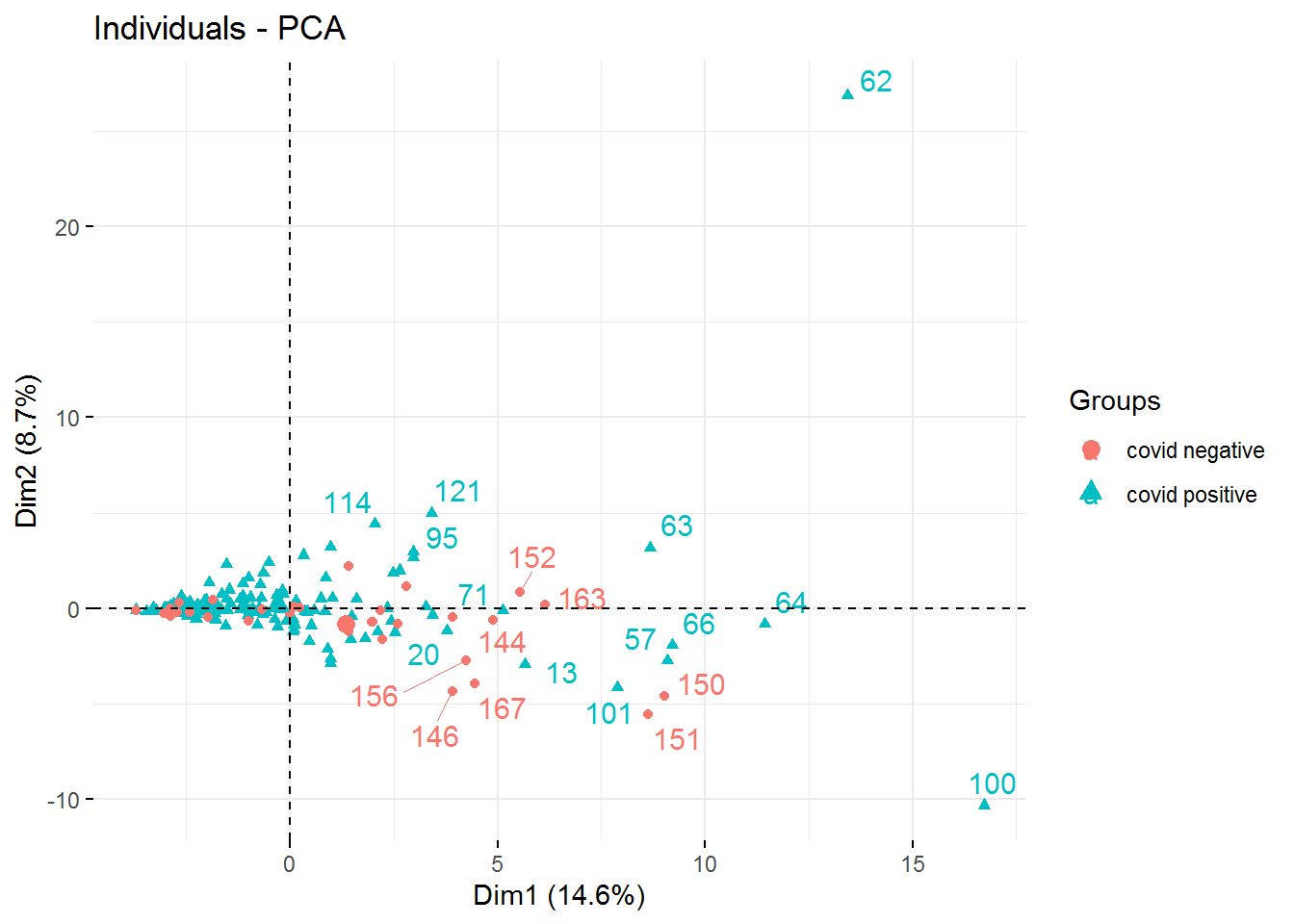

Supplement: Supplementary file 1 [file pathogens-13-00953-s001.zip › Figure S1.PCA plot.COVID.png]

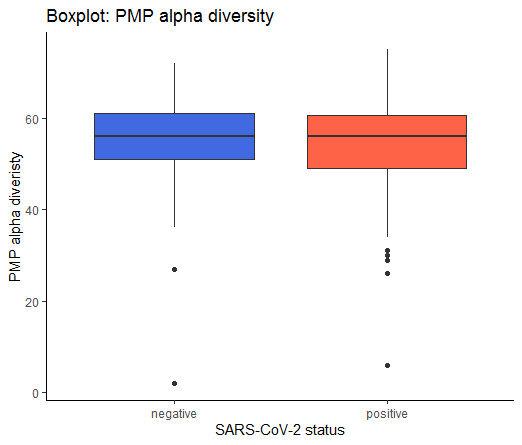

Supplement: Supplementary file 1 [file pathogens-13-00953-s001.zip › Figure S2. Boxplot .png]
